# Genistein prevents bone loss in type 2 diabetic rats induced by streptozotocin

**DOI:** 10.29219/fnr.v64.3666

**Published:** 2020-12-09

**Authors:** Rongrong Lu, Zicong Zheng, Yimin Yin, Zhuoqin Jiang

**Affiliations:** Department of Nutrition, School of Public Health, Sun Yat-Sen University, Guangzhou, People’s Republic of China

**Keywords:** genistein, diabetic osteoporosis, inflammation, osteoclasts, adipocytes

## Abstract

**Background:**

Diabetic osteoporosis has become a severe public health problem in the aging societies. Genistein has been reported to play an important role in preventing and treating metabolic diseases via its anti-inflammatory, antioxidant, anti-estrogenic, and estrogen-like functions.

**Objective:**

We aimed to investigate whether genistein exerts bone-protective effect on diabetic rats induced by 35 mg/kg streptozotocin (STZ) plus a 4-week high-fat diet.

**Design:**

Sprague–Dawley rats were randomly divided into four groups: (1) control group, (2) type 2 diabetes mellitus (T2DM) model group, (3) T2DM with 10 mg/kg genistein, and (4) T2DM with 30 mg/kg genistein. After an 8-week treatment with genistein, the femurs, tibias, and blood were collected from all rats for further analysis.

**Results:**

Genistein at 10 mg/kg showed little effect on diabetic osteoporosis, whereas genistein at 30 mg/kg significantly improved glucose and bone metabolisms compared with diabetic rats. Our results showed that 30 mg/kg genistein significantly increased bone mineral density, serum osteocalcin, and bone alkaline phosphatase. Genistein also effectively lowered fasting blood glucose, tartrate-resistant acid phosphatase 5b, tumor necrosis factor-α, interleukin-6, and numbers of adipocytes and osteoclasts. Compared with the T2DM group, protein levels of receptor activator of nuclear factor κB ligand (RANKL) and peroxisome proliferator-activated receptor-γ (PPAR-γ) were decreased, while protein levels of osteoprotegerin (OPG), β-catenin, and runt-related transcription factor 2 (Runx-2) were increased after genistein intervention.

**Conclusion:**

Genistein could effectively improve abnormal bone metabolism in STZ-induced diabetic rats; the underlying molecular mechanisms might be related to OPG/RANKL, PPAR-γ, and β-catenin/Runx-2 pathways.

## Popular scientific summary

- Type 2 diabetic rats induced by high-fat diet and low-dose streptozotocin showed decreased bone density and impaired bone microstructure.
- Genistein at a dosage of 30 mg/kg can improve abnormal bone metabolism in diabetic rats, which is probably related to its hypoglycemic and anti-inflammatory effects. The underlying molecular mechanisms might be related to protein levels of osteoprotegerin/receptor activator of nuclear factor κB ligand, peroxisome proliferator-activated receptor-γ, and β-catenin/Runx-2 pathways.

Based on the International Diabetes Federation, 451 million people aged 18–99 years were diagnosed with diabetes worldwide in 2017, and it was estimated that there would be nearly 693 million people with diabetes globally by 2045 (1). Osteoporosis causes more than 8.9 million fractures every year worldwide, almost one case every 3 sec (2). Both diabetes and osteoporosis cause serious threats to people’s health and life quality, especially for the elderly, which has become an important public health problem in the aging societies. In 1948, Albright et al. found that diabetes was related to osteoporosis and proposed the concept of diabetic osteoporosis (3). Diabetic osteoporosis, one of the severe complications of diabetes mellitus, is a systemic endocrine metabolic osteopathy, resulting in increased risk of bone-fragility-related fractures. Also, numerous researches have indicated that the incidence of both osteoporosis and fracture in type 2 diabetic patients is significantly higher than that in non-diabetic people (4–6).

Existing studies have shown that chronic hyperglycemia, oxidative stress, insulin resistance, advanced glycation end products, insulin-like growth factor-1, the use of hypoglycemic drugs, and other factors may play an important role in the pathophysiological process of diabetic osteoporosis (7–9). In type 2 diabetes mellitus (T2DM), long-term hyperglycemia and enhanced inflammatory cytokines have detrimental effects on bone metabolism, causing bone loss ultimately (10). On the one hand, hyperglycemia and increased inflammation lead to an increase in receptor activator of nuclear factor κB ligand (RANKL) and a decrease in osteoprotegerin (OPG), which contribute to greater bone resorption (11, 12). RANKL, a member of the tumor necrosis factor superfamily, could increase bone resorption through stimulating osteoclast proliferation and differentiation; on the contrary, OPG inhibits osteoclast maturation to reduce bone resorption (13, 14). On the other hand, diabetes-enhanced inflammation and hyperglycemia could induce apoptosis of mature osteoblasts and promote adipogenesis from mesenchymal stem cells, resulting in reduced osteoblast formation and differentiation, the underlying molecular mechanisms of which might be related to the decreased runt-related transcription factor 2 (Runx-2) and activated peroxisome proliferator-activated receptor-γ (PPAR-γ) (10, 15). Thus, both high blood glucose and inflammatory levels are associated with bone loss in T2DM. If they were controlled at the same time, diabetic osteoporosis could be protected against. At present, the main therapies for osteoporosis are antiresorptive drugs, osteoanabolic drugs, calcium, and vitamin D, which have no effect on high blood glucose and inflammatory response. In view of this problem, it is imperative to find an effective substance with hypoglycemic and anti-inflammatory effects to treat diabetic osteoporosis.

Genistein is one of the most abundant soy isoflavones, with anti-inflammatory, antioxidant, anti-osteoporosis, and hypoglycemic properties (16), which are rich in soybeans and soy-derived products (17). As a phytoestrogen, numerous studies have focused on that genistein prevents bone loss and improve bone health through estrogen receptor pathway in both postmenopausal women and ovariectomized animals (18). Recently, an epidemiological study suggested that soy-containing isoflavones could reduce bone resorption significantly in men with T2DM, which correlated with an improvement of glycemic control (19). A small number of animal studies reported that genistein could enhance bone health of animals with T2DM and increase the bone strength (20, 21). However, current evidences are limited to confirm the positive effects of genistein on diabetic osteoporosis, and the underlying molecular mechanism has not been elucidated yet.

At present, the animal models of type 2 diabetic osteoporosis are mainly divided into three categories: induced type 2 diabetes model, spontaneous type 2 diabetes model, and transgenic/knockout type 2 diabetes model (22). Studies have found that the diabetic rat induced by a high-fat (HF) diet and low-dosage streptozotocin (STZ) was a common model of type 2 diabetes. Rats with HF and high-glucose diet were induced to impaired glucose tolerance and insulin resistance, and low-dose STZ could specifically damage a small number of pancreatic islet cells (23, 24). In recent years, many scholars have used this model to study type 2 diabetic osteoporosis, in which diabetic rats appeared to have abnormal bone metabolism and severe bone loss (25–27). Thus, the present study aims to investigate the effect of genistein on bone metabolism in diabetic rats induced by a low dosage of STZ plus HF diet *in vivo*.

## Materials and methods

### Experimental animals and treatment

Seventy male Sprague–Dawley rats, weighing about 180–230 g, were purchased from the Experimental Animal Center of Guangdong Province, China, and housed in the specific pathogen free (SPF) animal room maintained at 22–26°C with 60–80% humidity and regular 12-h light–dark cycles (light time: 07:00–19:00). All procedures and protocols were supported by the Animal Experimental Ethics Committee of School of Public Health, Sun Yat-Sen University, China (permission number: 2018-010).

After a week of adaptive feeding, 70 rats were randomly divided into two groups according to their body weight: 10 rats were in the control (CON) group and fed with a normal diet consisting of 12 kcal% fat, 68 kcal% carbohydrates, 20 kcal% protein, and 3.62 kcal/g of food; the remaining 60 rats in the HF group were fed with an HF diet consisting of 37 kcal% fat, 46 kcal% carbohydrates, 17 kcal% protein, and 4.40 kcal/g of food. The normal diet and HF diet were purchased from Guangdong Medical Laboratory Animal Center (Guangzhou, China).

After 4 weeks of corresponding diet administration separately, rats in the HF group were intraperitoneally injected with 35 mg/kg body weight STZ (Sigma, USA) to induce T2DM; rats in the control group were injected with citrate buffer as a solvent comparison. After 72 h of STZ injection, rats were fasted for 8 h, but water was allowed. Tail venous blood was harvested to measure fasting blood glucose (FBG) and fasting insulin. Then, an oral glucose tolerance test (OGTT) was conducted after the rats received 50% aqueous glucose solution via oral gavage. Animals with FBG more than 11.1 mmol/L were considered as diabetic rats (28). The values of homeostasis model assessment of insulin resistance (HOMA-IR) were calculated from fasting glucose and insulin to measure the levels of insulin resistance, the formula of which was as follows: HOMA-IR = FBG (mmol/L) × fasting insulin (mU/L)/22.5 (29).

After that, there were four groups of rats in our study: (1) control group (CON, *n* = 10); (2) T2DM model group (T2DM, *n* = 11); (3) T2DM with 10 mg/kg body weight genistein (Sigma, USA), intragastrically (T2DM + GEN10, *n* = 11); and (4) T2DM with 30 mg/kg body weight genistein, intragastrically (T2DM + GEN30, *n* = 11). Rats in the CON and T2DM groups were both administered with 30% dimethyl sulfoxide (DMSO, vehicle control); rats in the T2DM + GEN10 and T2DM + GEN30 groups were given 10 mg/kg genistein and 30 mg/kg genistein by gavage, respectively. All rats were free to have food and water and were then treated with genistein or 30% DMSO for 8 weeks. Food intakes were recorded every day, and body weights were measured once a week.

### Sample collection and dual-energy X-ray absorptiometry

At the end of the experimental period, the rats were anesthetized with 3% pentobarbital sodium solution (0.2 mL/100 g) after 8 h of fasting. After anesthetizing, bone mineral density (BMD) and bone mineral content (BMC) of whole-body and spine were measured by dual-energy X-ray absorptiometry (Hologic, Boston) before sacrifice.

Blood was collected from the abdominal aorta rapidly and stood at room temperature for 30 min; then serum was separated by centrifugation (3,000 rpm, 10 min, 4°C) and saved at −80°C until further analysis. Bilateral femurs and tibias were rapidly separated and weighed together after washing with pre-cold phosphate-buffered saline. Then femurs from different animals were fixed in 4% paraformaldehyde (Beyotime, China) for histological examination and micro-computed tomography (μ-CT); the rest were frozen at −80°C for the subsequent experiments.

### Bone microarchitecture measurement with μ-CT

The isolated femurs were scanned with μ-CT (μ-CT 100, SCANCO Medical, Switzerland) according to its recommended guidelines and the procedures of Cao et al. (30). Trabecular parameters were calculated using the software (Ray V4.0-4, Switzerland), including trabecular bone volume fraction (BV/TV), trabecular number (Tb.N), trabecular thickness (Tb.Th), trabecular separation (Tb.Sp), and structure model index (SMI).

### Histology and tartrate-resistant acid phosphatase staining

Hematoxylin–eosin (H&E) and tartrate-resistant acid phosphatase (TRAP) staining were performed according to Rivoira et al. (31). The morphological changes of bone tissue were observed under an inverted fluorescence microscope (Nikon, Japan) and then the digital microphotographs were obtained. Numbers of adipocytes (adipocytes/mm^2^) and TRAP-positive osteoclasts (osteoclasts/mm^2^) were counted using Image Pro Plus 6.0 software (Media Cybernetics, Inc., USA).

### Biochemical investigations

FBG was determined using commercial kits (Servicebio, China) using enzymatic colorimetric method. OGTT test was performed using a one-touch glucometer (Select Simple, Johnson&Johnson, Shanghai, China), and the area under the glucose curve (AUC) was calculated from the blood glucose concentrations measured at 0, 30, 60, 90, and 120 min. Fasting insulin (CUSABIO, China), osteocalcin (OCN) (Nanjing Jiancheng, China), bone alkaline phosphatase (BALP) (CUSABIO, China), tartrate-resistant acid phosphatase 5b (TRACP-5b) (CUSABIO, China), TNF-α (Nanjing Jiancheng, China), and interleukin-6 (IL-6) (Nanjing Jiancheng, China) were measured using enzyme-linked immunosorbent assay in accordance with the manufacturer’s instructions.

### Western blotting

The fresh bone tissues were fully soaked in pre-cooled normal saline and washed with distilled water to remove blood and red blood cells. The tissues were then cut up, weighed, and put into a mortar containing liquid nitrogen, which was ground into powder. Hundred milligram of bone powder was added into a centrifuge tube with 1 mL protein extraction reagent containing 1 mmol phenylmethylsulfonyl flouride (Beyotime, China). This was then homogenized in an ice bath for 30 min and oscillated on the swirl mixer every 5 min for 20 sec. The supernatant was harvested after centrifugation at 12,000 rpm for 15 min at 4°C. The levels of total protein were detected by bicinchoninic acid assay with commercial kits (Beyotime, China) and degenerated with loading buffer for 5 min at 100°C. The specimens with the same content of protein (50 μg) were separated through sodium dodecyl sulfate–polyacrylamide gel electrophoresis and electrotransferred to polyvinylidenedifluoride membranes (PVDF; Millipore, USA). The membranes were then incubated for 2 h with tris-buffered saline with Tween-20 blocking solution containing 5% skimmed milk and overnight with the primary antibodies of β-actin (1:1,000; No. ab8227; Abcam, UK), OPG (1:3,000; No. ab73400; Abcam, UK), RANKL (1:1,000; No. 23408-1-AP; Proteintech Group, Inc., USA), PPAR-γ (1:1,000; No. 16643-1-AP; Proteintech Group, Inc., USA), β-catenin (1:5,000; No. ab32572; Abcam, UK), and Runx-2 (1:3,000; No. ab76956; Abcam, UK). Thereafter, the brands were washed three times with TBS and incubated with goat anti-rabbit secondary antibodies (Beyotime, China) for 2 h at room temperature. The brands were scanned using an electrochemiluminescence (ECL) detection system after adding BeyoECL Plus (Beyotime, China), and their densities were analyzed using Image J software (version 1.43, USA).

### Statistical analysis

Statistical analyses were performed with SPSS 19.0, and the results were expressed as means ± standard deviation (SD). One-way analysis of variance was used to compare data between groups. Bonferroni or Kruskal–Wallis *H* was used in the comparison between the two groups. Differences were considered statistically significant at *P* < 0.05.

## Results

### The result of T2DM modeling

**Fig. 1:**
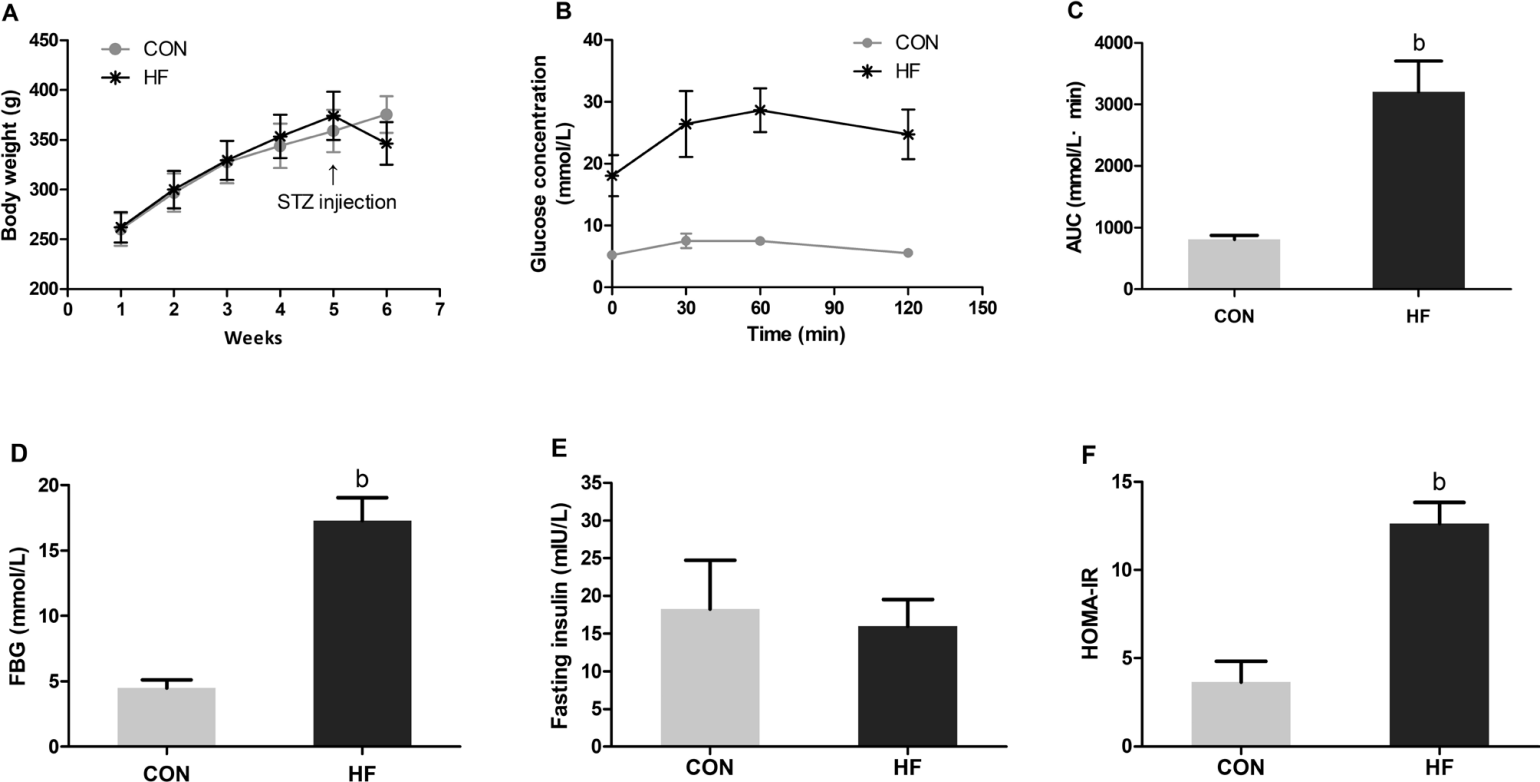
The levels of body weight (A), OGTT (B), AUC (C), FBG (D), fasting insulin (E), and HOMA-IR (F) in the CON and HF groups after STZ injection. Values are expressed as mean ± SD. ^a^*P* < 0.05, ^b^*P*< 0.01 compared with CON. CON: the control group (*n* = 10); HF: the high-fat group (*n* = 33).

Thirty-three rats in the HF group were successfully induced with T2DM, showing abnormal glucose tolerance and insulin resistance. As illustrated in Fig. 1A, the body weight of all rats showed an increasing trend every week before STZ injection; after STZ injection, the body weight in the CON group continued to increase, whereas body weight in the HF group declined rapidly. Figure 1C and D shows that both FBG and AUC in the HF group were much higher than those in the CON group after STZ injection (*P* < 0.05). As for the fasting insulin levels, no significant difference was observed between the CON and HF groups (Fig. 1E); however, the HOMA-IR values in the HF group were significantly higher than those in the CON group (Fig. 1F).

### Effect of genistein on general situation of diabetic rats

**Fig. 2:**
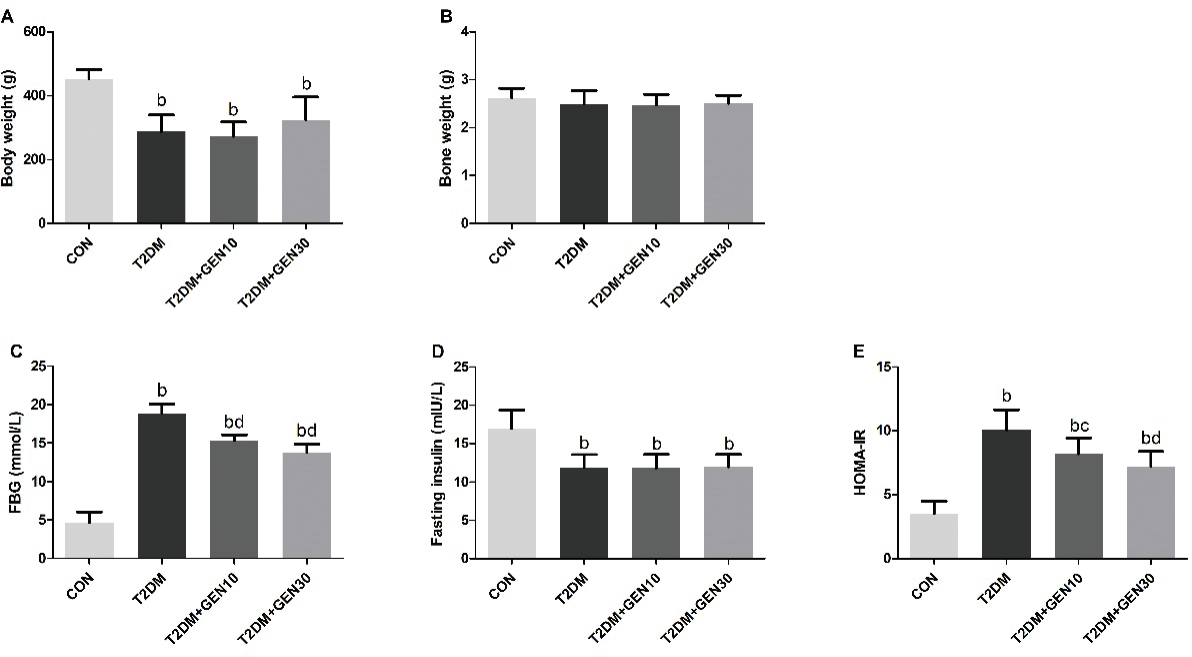
The levels of body weight (A), bone weight (B), FBG (C), fasting insulin (D), and HOMA-IR (E) in different groups after genistein intervention. Bone weight (g) = tibia weight (g) + femur weight (g). Values are expressed as mean ± SD, *n* = 10 or 11. ^a^*P* < 0.05, ^b^*P* < 0.01 compared with CON; ^c^*P* < 0.05, ^d^*P* < 0.01 compared with T2DM.

Food and water intakes in the T2DM group were more than those in the CON group; however, both low-dose and high-dose genistein interventions did not significantly change food and water consumptions compared with the T2DM group (data not shown). At last, the body weight of rats in the T2DM group was lower than that in the CON group (*P* < 0.05); 30 mg/kg genistein could increase the body weight of diabetic rats, while the difference was not statistically significant (*P* > 0.05; Fig. 2A). Also, there was no difference in bone weight among all groups (*P* > 0.05; Fig. 2B).

### Effect of genistein on FBG, fasting insulin, and HOMA-IR levels

Compared with the CON group, the FBG in the T2DM group was increased by fourfold, whereas diabetic rats administered with low- and high-dose genistein decreased the FBG by 18.74 and 26.75%, respectively, compared with the T2DM group (*P* < 0.05; Fig. 2C). Similarly, a remarkable increase of HOMA-IR was observed in the T2DM group, whereas genistein treatment groups lowered the HOMA-IR in a dose-dependent manner (*P* < 0.05; Fig. 2E). Fasting insulin was significantly lowered in the three diabetic groups compared with the CON group (Fig. 2D).

### Genistein improved BMD and BMC levels of diabetic rats

**Fig. 3:**
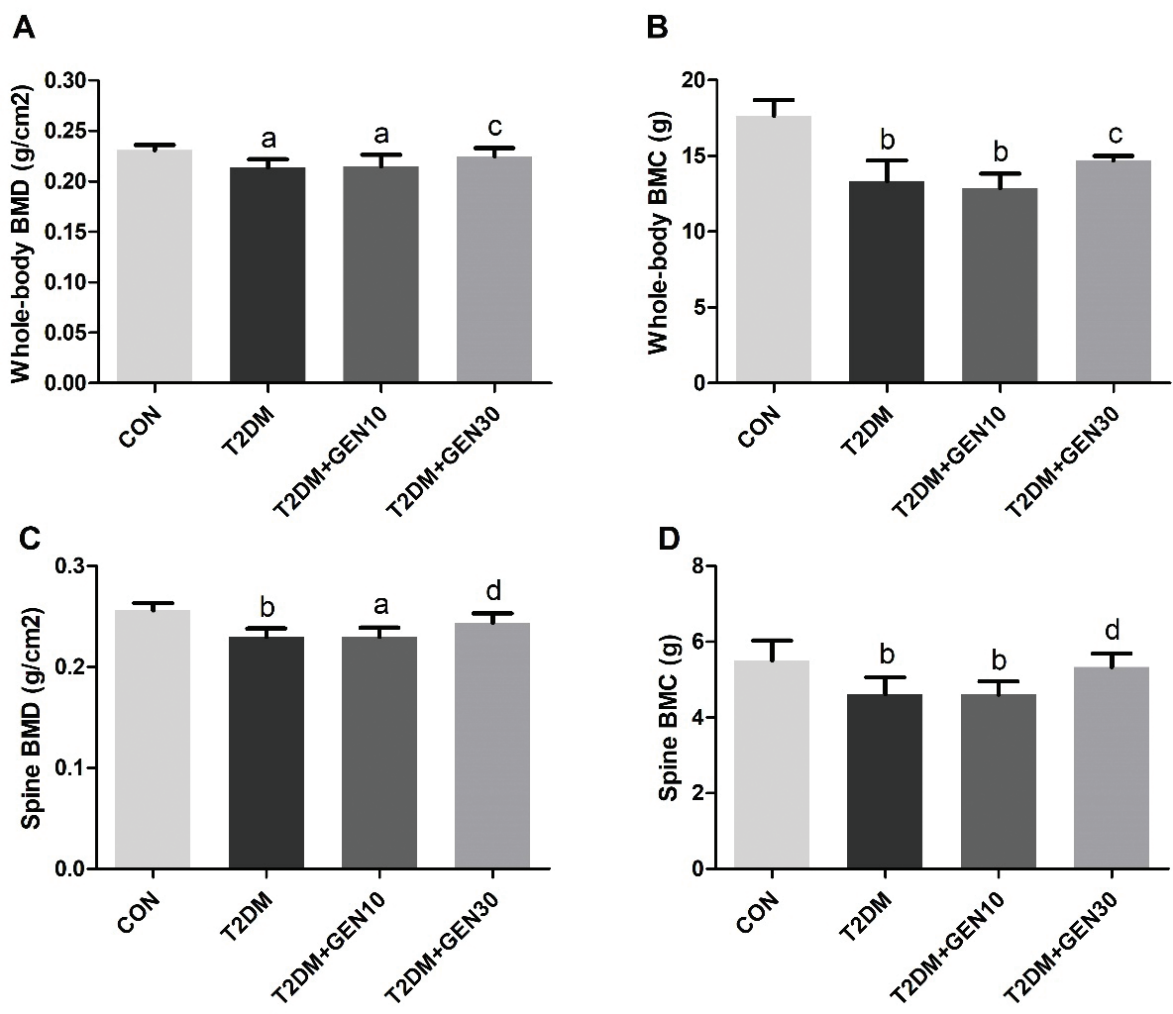
Bone mineral density (BMD) and bone mineral content (BMC) of whole body (A, B) and spine (C, D). Values are expressed as mean ± SD, *n* = 10 or 11. ^a^*P* < 0.05, ^b^*P* < 0.01 compared with CON; ^c^*P* < 0.05, ^d^*P* < 0.01 compared with T2DM.

With the final body weight as a covariate, the BMD and BMC of the rats in each group were analyzed by covariance. As illustrated in Fig. 3, both BMD and BMC levels of whole-body and spine in the T2DM group were significantly lower than those in the CON group (*P* < 0.05), which suggested that diabetic rats developed bone loss. The results of those in the T2DM + GEN10 group were similar to the T2DM group (*P* > 0.05), showing no significant improvement. However, rats administered with high-dose genistein notably restored BMD and BMC levels of both whole-body and spine compare with the T2DM group (*P* < 0.05), increasing by 5.14, 10.26, 6.09, and 15.67%, respectively. Interestingly, although the BMD and BMC levels of whole-body and spine did not reach the levels of those in the CON group, there was no significant difference between the CON and T2DM + GEN30 groups (*P* > 0.05). These results indicated that genistein at a concentration of 30 mg/kg could prevent bone loss in STZ-induced diabetic rats.

### Genistein improved impaired bone microarchitecture of diabetic rats

**Fig. 4:**
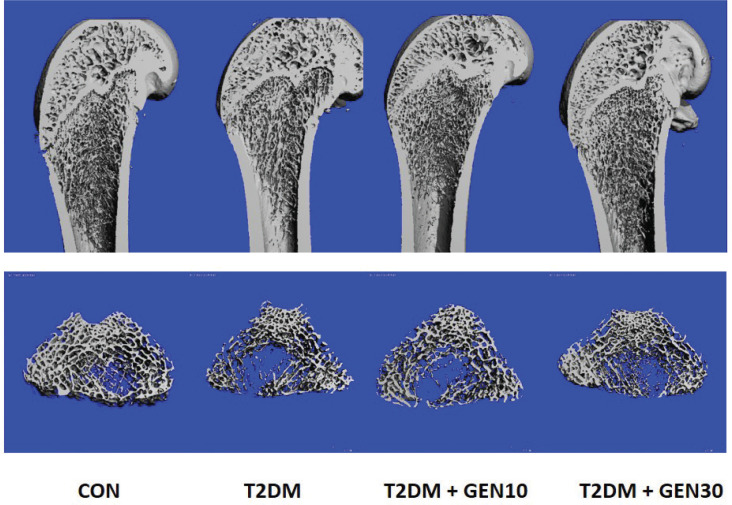
The representative longitudinal images of the distal femur and three-dimensional images of bone trabecula were obtained by μ-CT in four groups at the end of the experiment.

The longitudinal images of distal femurs and three-dimensional images of bone trabecula from four groups were obtained using μ-CT (Fig. 4). The results of trabecular microarchitectural parameters (including TV/BV, Tb.Th, Tb.N, and Tb.Sp) are demonstrated in Table 1. In comparison with the CON group, rats in the T2DM group showed a remarkable decrease in TV/BV and Tb.N, as well as a significant increase in Tb.Sp and SMI. After genistein treatment, the levels of TV/BV and Tb.N were significantly increased and the levels of Tb.Sp and SMI were significantly decreased compared with the T2DM group. Compared with the T2DM group, the levels of BV/TV and Tb.Th in the T2DM + GEN30 group were increased by 23.88 and 27.05%, while the levels of Tb.Sp and SMI in the T2DM + GEN30 group were decreased by 23.53 and 13.96%, respectively. It was noteworthy that Tb.N and Tb.Sp showed no statistical difference between the T2DM + GEN30 and the CON groups (*P* > 0.05). However, there was no significant difference in Tb.Th between the four groups (*P* > 0.05).

**Table 1:** Femur bone microarchitecture parameters among groups

Index	CON	T2DM	T2DM + GEN10	T2DM + GEN30
TV/BV (%)	22.05 ± 0.31	13.44 ± 0.36^b^	15.38 ± 0.24^bd^	16.65 ± 0.36^bd^
Tb.Th (mm)	0.073 ± 0.008	0.064 ± 0.005	0.066 ± 0.006	0.066 ± 0.007
Tb.N (1 mm^-1^)	3.87 ± 0.10	2.92 ± 0.10^b^	3.28 ± 0.11^bd^	3.71 ± 0.12^d^
Tb.Sp (mm)	0.24 ± 0.01	0.34 ± 0.02^b^	0.30 ± 0.03^bd^	0.26 ± 0.01^d^
SMI	1.43 ± 0.16	2.22 ± 0.12^b^	2.06 ± 0.09^b^	1.91 ± 0.10^bc^

Values are expressed as mean ± SD, *n* = 10 or 11. ^a^*P* < 0.05

b *P* < 0.01 compared with CON

c *P* < 0.05

d *P* < 0.01 compared with T2DM.

### Genistein decreased the numbers of adipocytes and TRAP-positive osteoclasts

**Fig. 5:**
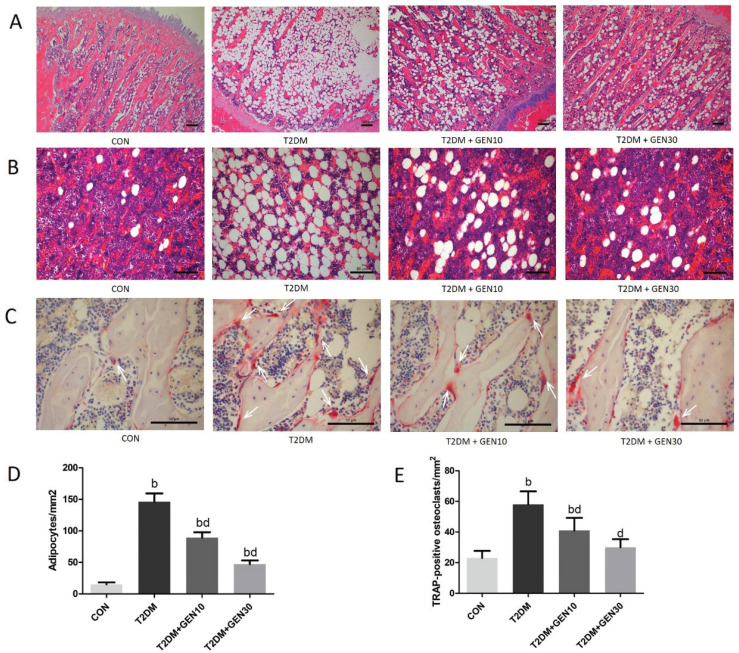
The left femurs were removed after 8 weeks of genistein or vehicle treatment. The histomorphology of bone tissues with H&E and TRAP staining was observed under a microscope. (A) Representative H&E staining of femur sections from each group. (B) Bone marrow adipocytes of rats with H&E staining in each group. (C) Representative TRAP staining of femur sections from each group. TRAP-positive osteoclasts with red cytoplasm and blue nucleus are indicated with white arrows. (D) Quantification of the number of adipocytes per square millimeter in left femurs. (E) Quantification of the number of TRAP-positive osteoclasts per square millimeter in left femurs. Values are expressed as mean ± SD, *n* = 10 or 11. ^a^*P* < 0.05, ^b^*P* < 0.01 compared with CON; ^c^*P* < 0.05, ^d^*P* < 0.01 compared with T2DM.

Figure 5A shows the H&E staining of distal femoral metaphysis in rats. In the T2DM group, the bone trabecular became thinner with poor morphological structure, and the trabecular separation increased, indicating that diabetic rats induced by an HF diet and STZ showed a deteriorated bone trabecular microstructure. Compared with the T2DM group, genistein treatment effectively improved the bone structure and restored the bone histomorphology, especially in the T2DM + GEN30 group.

Rats in the T2DM group showed much higher numbers of adipocytes (Fig. 5B, D) and TRAP-positive osteoclasts (Fig. 5C, E) than those in the CON group (*P* < 0.05); however, genistein reduced both adipocyte and osteoclast numbers dose dependently (*P* < 0.05). Compared with the T2DM group, the numbers of adipocytes and TRAP-positive osteoclasts in the T2DM + GEN30 group were decreased by 67.81 and 48.28%, respectively, although the levels of adipocytes did not recover to the control ones.

### Effects of genistein on serum biochemistry

**Fig. 6:**
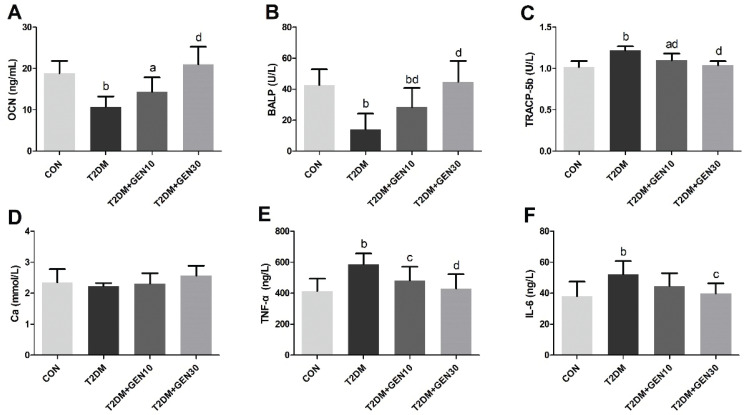
Serum OCN (A), BALP (B), TRACP-5b (C), Ca (D), TNF-α (E), and IL-6 (F) levels in each group. Values are expressed as mean ± SD, *n* = 10 or 11. ^a^*P* < 0.05, ^b^*P* < 0.01 compared with CON; ^c^*P* < 0.05, ^d^*P* < 0.01 compared with T2DM.

Serum OCN and BALP were characteristic markers of bone formation (32), and TRACP-5b was a marker of bone resorption (33), which could be commonly used to evaluate osteoblastic and osteoclastic activities. Compared with the CON group, serum OCN and BALP activities in the T2DM group were significantly reduced (*P* < 0.05; Fig. 6A, B); however, genistein administration inhibited the reductions of OCN and BALP. Compared with the T2DM group, serum OCN and BALP were increased by 95.36 and 217.57%, respectively, in the T2DM + GEN30 group. Furthermore, we found that TRACP-5b activity showed a remarkable increase in the T2DM group compared with the CON group, and then it was decreased by genistein (*P* < 0.05; Fig. 6C). Genistein at the dosage of 30 mg/kg significantly decreased the TRACP-5b activity, which was close to that in the CON group. As for the levels of serum Ca, there was no difference between the four groups (*P* > 0.05; Fig. 6D).

Serum TNF-α and IL-6, as an indicator of inflammatory response status, were elevated obviously in the T2DM group, compared with the CON group (*P* < 0.05; Fig. 6E, F). Nevertheless, genistein intervention reversed the elevated TNF-α and IL-6 levels significantly (*P* < 0.05). Compared with the T2DM group, serum TNF-α and IL-6 in the T2DM + GEN30 group were decreased by 26.90 and 23.76%, respectively.

### Protein levels of OPG, RANKL, PPAR-γ, β-catenin, and Runx-2 in the bone

**Fig. 7:**
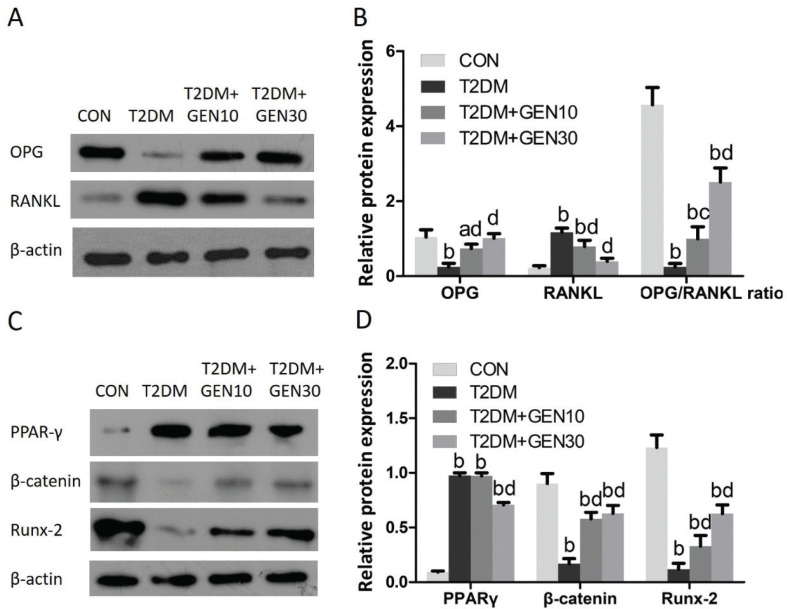
Genistein regulates the protein expressions of OPG, RANKL, PPAR-γ, β-catenin, and Runx-2 in the right femur. (A) Protein expressions of OPG, RANKL, and β-actin were shown using Western blotting images. (B) Relative protein levels were normalized to β-actin. (C) Protein expressions of PPAR-γ, β-catenin, Runx-2, and β-actin were shown using Western blotting images. (D) Relative protein levels were normalized to β-actin. ^a^*P* < 0.05, ^b^*P* < 0.01 compared with CON; ^c^*P* < 0.05, ^d^*P* < 0.01 compared with T2DM.

As shown in Fig. 7A and B, the OPG protein level and the OPG/RANKL ratio in the T2DM group were both significantly lower than those in the CON group (*P* < 0.05). However, they were increased in genistein-treated groups compared with the T2DM group (*P* < 0.05). The RANKL protein level in the T2DM group was dramatically increased compared with the CON group (*P* < 0.05), whereas genistein treatment reduced it compared with the T2DM group (*P* < 0.05).

As demonstrated in Fig. 7C and D, PPAR-γ protein level in the T2DM group was much more than that in the CON group (*P* < 0.05). The elevated PPAR-γ protein level in diabetic rats was decreased by high-dose genistein intervention, but it didn’t decline to the control level. With regard to β-catenin and Runx-2 protein levels, they were markedly decreased in diabetic rats compared with the CON group (*P* < 0.05). Genistein treatment increased the β-catenin and Runx-2 protein levels, but they were not able to return to the control levels.

## Discussion

It is widely known that genistein plays an important role in preventing and treating obesity, cancer, osteoporosis, diabetes and other metabolic syndromes via its anti-inflammatory, antioxidant, and estrogenic effects (34). However, the effect of genistein on bone metabolism in diabetic rats rather than in ovariectomized rats was first explored in an *in vivo* study. In our study, we found 30 mg/kg genistein could improve bone loss in STZ-induced diabetic rats.

In our study, the model group rats showed higher FBG, AUC, and HOMA-IR values after STZ injection and also showed symptoms of polyphagia, polyuria, and weight loss gradually, indicating that we successfully induced T2DM rat models with hyperglycemia and insulin resistance. Previous studies reported that T2DM rat models had either lower, higher, or same levels of insulin compared with the controls, which may be associated with early or late stages of type 2 diabetes. Serum insulin in the early stage of T2DM was at a higher level, whereas the late stage appeared similar to the control or lower insulin levels (23, 25). In our study, diabetic rats showed no significant change in fasting insulin after STZ injection; fasting insulin in the three diabetic groups decreased significantly after the experiment, which may be related to the stage of T2DM and disease progression. The T2DM rat model we induced probably represented the late stage of type 2 diabetes, and serum insulin was deficient along with the disease progress. Fortunately, the FBG and HOMA-IR in genistein treatment groups were significantly decreased, indicating that genistein could improve the blood glucose to some extent without restoring the insulin level in diabetic rats.

BMD, bone histomorphology, and bone turnover markers were utilized to assess bone quality, bone strength, and fracture risk (35, 36). At present, μ-CT was considered as a gold standard for measuring bone microarchitectural parameters to predict bone loss and bone structure, such as TV/BV, Tb.Th, Tb.N, and Tb.Sp. Bone turnover markers are divided into two groups: bone resorption markers, such as TRACP-5b, and bone formation markers, such as OCN and BALP, which reflect the activities of osteoblasts and osteoclasts, respectively (37). In our study, T2DM rat models showed reduced BMD, decreased OCN and BALP, increased TRACP-5b, increased numbers of bone marrow adipocytes and TRAP-positive osteoclasts, as well as a deteriorated bone microstructure, which was consistent with previous studies (25, 38). Notably, genistein at a daily dosage of 30 mg/kg markedly improved these changes. In addition, both H&E staining and μ-CT indicated that genistein had a positive role in improving bone microarchitecture and preventing bone loss.

Osteoblasts and osteoclasts regulated the balance between bone formation and bone absorption through the OPG/RANKL/RANK system. Osteoblasts and bone marrow stromal cells express RANKL, which can bind with the RANK on osteoclasts to stimulate osteoclast maturation and increase bone resorption. Simultaneously, osteoblasts also secrete OPG, which competitively combines with RANK and inhibits formation and maturation of osteoclasts (39). Numerous studies showed that hyperglycemia and enhanced inflammation caused by diabetes might lead to an increased RANKL/OPG ratio or affect osteoblasts through other mechanisms to aggravate bone resorption, and ultimately increase the fracture risk (10, 40–43). *In vitro* studies showed that genistein or daidzein could decrease bone resorption and promote bone formation by regulating the expression of OPG and RANKL as well as suppressing TNF-α and IL-6, so as to prevent osteoporosis (44, 45). In this study, our results showed that 30 mg/kg genistein could reduce blood glucose and inflammation, which is one of important reasons for its anti-diabetic osteoporosis. Interestingly, genistein also increased OPG protein level and OPG/RANKL ratio, as well as decreased RANKL protein level and the number of TRAP-positive osteoclasts in bone tissue, which might be one of the important mechanisms to protect against STZ-induced diabetic osteoporosis. In addition, it was equal for adipocytes or osteoblasts to be derived from multipotential mesenchymal stem cells in the bone marrow. Wnt/β-catenin pathway modulates mesenchymal stem cell differentiation, and osteoblastogenesis is activated by downregulating PPAR-γ and elevating Runx-2 (12). Our study found that a 30-mg/kg genistein treatment for 8 weeks increased β-catenin and Runx-2 protein levels and decreased PPAR-γ protein level and the number of bone marrow adipocytes in diabetic rats, which suggested the bone-protective effect of genistein in diabetic rats might be related to the inhibition of PPAR-γ and stimulation of β-catenin and Runx-2.

In our literature, the intervention dosages of genistein (10 and 30 mg/kg) were referred to the previous literature, in which 3–50 mg/kg genistein treatment every day could protect against ovariectomized-induced bone loss (46). The daily intake of 10 mg/kg genistein in rats was probably equivalent to the genistein daily intake in a soy-enriched diet in humans (47, 48). Finally, our study demonstrated that genistein at a dosage of 30 mg/kg could significantly alleviate blood glucose and prevent bone loss in STZ-induced diabetic rats, suggesting the effective dosage of genistein is higher than the average daily intake of most people. Thus, consumption of soy and its products should be encouraged and even concentrated genistein products or its supplements might be required.

However, there were some limitations in this research. The data we collected for analysis was limited, because the number of experimental animals was small and the biomechanical test was not conducted. More importantly, it is necessary to prove the direct effects of genistein on osteoblasts and osteoclasts from an *in vitro* study and to provide more robust evidence to identify how the molecular mechanisms work. There may also be many complicated signaling pathways involved in the result, including the estrogen receptor mediating mechanism (49) and wnt pathways (50), so further studies are still essential to elucidate other underlying mechanisms and verify how they work.
